# Advocacy training for young family doctors in primary mental health care: a report and global call to action

**DOI:** 10.3399/BJGPO.2021.0163

**Published:** 2022-02-09

**Authors:** Sina Haj Amor, Tamica Daniels-Williamson, Krystle Fraser-Barclay, Christopher Dowrick, Emma C Gilchrist, Stephanie Gold, Larry A Green, Sarah Hemeida, Amanda Howe, Bonnie Jortberg, Cindy Lam, Henry Owuor, Helen Page, Sankha Randenikumara

**Affiliations:** 1 Faculty of Medicine of Tunis, Health District of Carthage, Tunis, Tunisia; 2 Family Medicine Residency Programme Guyana, Georgetown, Guyana; 3 Family Medicine Residency Programme, Institute of Health Science Education, Georgetown Public Hospital Corporation, Georgetown, Guyana; 4 WONCA Working Party for Mental Health, and Primary Care and Mental Health, University of Liverpool, Liverpool, UK; 5 Eugene S Farley, Jr. Health Policy Center, University of Colorado Anschutz Medical Campus, Aurora, Colorado, USA; 6 Norwich Medical School, University of East Anglia, Norwich, UK; 7 Family Medicine, University of Hong Kong, Ap Lei Chau, Hong Kong SAR; 8 Nyamira County Referral Hospital, Nyamira, Kenya; 9 Department of Primary Care and Mental Health, Institute of Psychology, University of Liverpool, Liverpool, UK; 10 The Family Health Clinic, Colombo, Sri Lanka

**Keywords:** Continuing professional development, Mental health (general), General practice, Family medicine, Primary healthcare

## Background

The World Health Organization (WHO) recognises the essential role of mental health in achieving health for all; its mental health action plan calls for more effective leadership for mental health and the provision of community-based, integrated care.^1^ However, integrating mental health care into primary care is a challenging, transformational change that requires more than clinical knowledge.^2^ It depends on strong advocacy, leadership, and change management: skills that can be learnt.^3,4^

## Project

**Figure 1.**
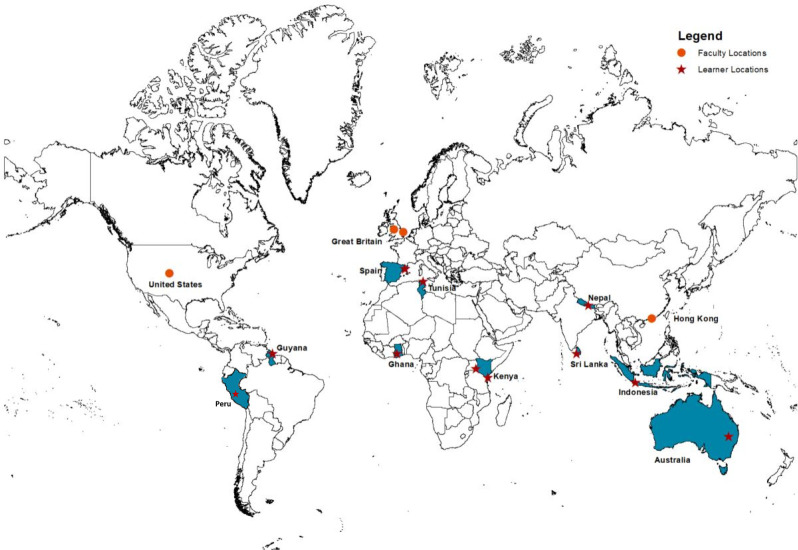
Map of learner and faculty countries.

The Farley Health Policy Centre (FHPC) partnered with the World Organization of Family Doctors (WONCA) to develop and pilot a global curriculum to enable learners to lead practice transformation and be empowered with policy-influencing skills to advocate for their patients, to promote and enhance primary care mental health. We recruited 12 young family doctors, of whom seven were women and ten were from low- and middle-income countries (LMICs), as shown in Figure 1.

The programme began with a survey of learners’ needs and aspirations, and an expectation that each would self-identify a practice transformation goal. Faculty and learners took part in a two-phase learning evaluation.

Funding from WONCA was provided for logistics and evaluation. A small stipend was offered to each learner on successful course completion. Faculty gave their time pro bono. The programme was conducted between March and October 2020. The evaluation process was approved by the University of Liverpool Health and Life Sciences Research Ethics Committee.

The learners were divided into two learning cohorts. Sessions were facilitated by two leaders and supported by four mentors. The educational content was delivered twice (to accommodate differing time zones) in six 90-minute monthly virtual sessions. The topics were:

- Introduction to mental health integration
- Leadership
- Team-based Care
- Quality Improvement
- Burnout and Resilience
- Advocacy

A seventh session was added at the end of the programme, to celebrate progress and award certification of completion.

Learners also developed individual projects to undertake in their own locality, with support and guidance from their cohort’s mentors and facilitators, and with feedback from other learners. The projects covered five broad goals:

- Educate other primary care doctors about integrated care, mental health conditions, and advocating for better mental health care (five learners)
- Improve team-based care to address mental health needs (three learners)
- Establish high quality systems of care to address mental health needs (two learners)
- Collate community resources for social prescribing (one learner)
- Increase patient awareness of mental health issues and available supports (one learner)

## Evaluation

### Participation

Learners demonstrated consistently high levels of participation, despite many varied pressures on their time: total participation rate across the education sessions was 96%.

### Initial needs and aspirations

Faculty guiding principles were to provide access to current knowledge and teach young family doctors how to transform their practice and lead change; they also aspired to pass on the mantle of mental health advocacy to emerging leaders. Learners’ guiding principles were to act as champions to improve mental health.

### Interim process evaluation

The learners’ initial expectations of the programme did not generally match faculty’s design of the course. They were happy with the online learning environment, found the facilitation of webinars excellent, and valued teaching materials. They wanted an informal, less US-centric first session. They valued proactive mentors and suggested mentors from LMICs might better understand their health care systems. They were concerned about the sustainability of their planned projects. They proposed a WhatsApp group to facilitate discussion and support between sessions. Faculty responded by modifying subsequent content, adding motivation techniques and capacity-building. A WhatsApp group was created and utilised for discussion between sessions.

### Final process evaluation

Learners were asked about their learning cohorts, mentoring, the impact of the pandemic, and key things they would take away from the pilot programme:

- Group cohesion. Learners felt it took some time to understand each other’s contexts but found their diversity of settings enriching: *’learning from other doctors’ experience doing a project is so empowering and inspiring*’.
- Mentorship. They felt their mentors were very experienced and provided good support, though some ’*could reach out a bit more’* and some offered *‘no support in later months*’.
- The impact of COVID was profound. All learners made incremental progress with their projects but some were unable to complete as planned. Meetings with other services and staff were restricted and patient care was affected.
- Increased confidence and skills. Learners described how ’*advocacy is an art that can be learnt*‘. The key content was *‘being intentional in performing leadership functions*’.
- This was a very resilient and optimistic group of clinicians. They modified their goals, seeing the pandemic as an opportunity to make changes for the better.
- Several learners still wanted further clinical skills training.

## Conclusion

Despite initiation in the midst of the COVID-19 pandemic, this pilot project was successfully implemented with remarkable commitment of participants. The structure and operations of the programme were confirmed to be feasible. The embedded evaluation provided important insights for improvement and affirmation that the programme was relevant, desired, and acceptable in different country environments. The learners formed their own community, finding their heterogeneity enriching. Several learners, already in positions of influence, recognised their ability to advocate for policy change.

This project has demonstrated a widespread appetite and ability among young family doctors to integrate mental health and primary care. The intervention was successful in equipping them to create systems changes to this end, and is reproducible in most countries. The lessons learnt are applicable in global level policies related to medical education and healthcare.

The next steps are to initiate a fully-funded rolling programme, and to scale up the global reach of this project. We are hopeful that this will enhance the health of populations and the professional satisfaction in the family doctors who participate.

Mental, emotional, and behavioural problems spare no country or community. There is a closable gap between what we know and what we do, and the people of the world are waiting for collaborative efforts to close it.

Every day, every family doctor sees large burdens of illness and need related to mental, emotional, and behavioural–emotional issues. If these patients’ needs are to be met, family doctors worldwide must integrate additional mental health services into their practices, and connect patients with relevant local resources. This is not a ‘tweak’ but a substantial change in practice. Primary care that responds to patients’ mental, emotional, and behavioural problems is feasible. It is also better, whole-person care.
